# Machine learning-based prediction of nasopharyngeal carcinoma risk: a clinical approach

**DOI:** 10.3389/fimmu.2025.1648648

**Published:** 2025-11-27

**Authors:** Wenhui Yang, Chengyan Zhou, Minzhong Tang, Zhiqiang Huang, Haiqing Zhu, Shangyang Li, Huipin Huang, Yujuan Liang, Wenting Pan, Yulin Yuan

**Affiliations:** 1 Department of Laboratory Medicine, The People’s Hospital of Guangxi Zhuang Autonomous Region, Nanning, Guangxi Zhuang Autonomous Region, China; 2 Department of Dermatology, The People’s Hospital of Guangxi Zhuang Autonomous Region, Nanning, Guangxi Zhuang Autonomous Region, China; 3 Key Laboratory of Nasopharyngeal Carcinoma Molecular Epidemiology, Wuzhou Red Cross Hospital, Wuzhou, Guangxi Zhuang Autonomous Region, China; 4 Department of Laboratory Medicine, The First People’s Hospital of Fangchenggang City, Fangchenggang, Guangxi Zhuang Autonomous Region, China; 5 Department of Laboratory Medicine, The People’s Hospital of Yongning District, Nanning, Guangxi Zhuang Autonomous Region, China

**Keywords:** nasopharyngeal carcinoma (NPC), NPC screening, predictive modeling, machine learning (ML), Epstein-Barr virus (EBV), logistic regression

## Abstract

**Background:**

Early screening and risk assessment of nasopharyngeal carcinoma (NPC) are essential for timely diagnosis and improved treatment outcomes. This study aimed to develop and evaluate predictive models using logistic regression and machine learning (ML) techniques to identify significant risk factors for NPC across various healthcare settings.

**Methods:**

A total of 569 participants were enrolled in the internal training and validation cohorts, and 160 were enrolled in the independent external validation cohort. Several Epstein-Barr virus (EBV)-related antibodies and serological and hematological markers were assessed to identify discriminatory features between NPC and non-NPC individuals. Feature selection was performed using least absolute shrinkage and selection operator (LASSO) regression, recursive feature elimination cross-validation (REFCV), and support vector machine recursive feature elimination cross-validation (SVMREFCV). The performance of nine machine learning (ML) models (logistic regression (LR), eXtreme Gradient Boosting (XGBoost), light gradient boosting machine (LightGBM), random forest (RF), AdaBoost, multilayer perceptron (MLP), decision tree (DT), gradient boosting decision tree (GBDT), and Gaussian Naïve Bayes (GNB)) was evaluated using the area under the curve (AUC), accuracy (ACC), sensitivity (SE), and specificity (SP) in both the training and validation cohorts. Model calibration was assessed using calibration plots and clinical utility was evaluated through decision curve analysis (DCA).

**Results:**

Five key predictors (nuclear antigen 1 immunoglobulin A (NTA1-IgA), viral capsid antigen immunoglobulin A (VCA-IgA), Rta protein immunoglobulin A (Rta-IgA), platelet (PLT) count, and lymphocyte (LM) count) were consistently identified across the three feature selection algorithms. The XGBoost model achieved the highest performance in the internal training (AUC = 0.999) and validation cohorts (AUC = 0.995); it also outperformed in the independent external validation cohort with an AUC of 0.956. Calibration and DCA for both internal and intendent external cohorts were then confirmed the strong clinical utility for the XGBoost model. An outline tool also enabled real-time NPC risk prediction based on the five selected biomarkers.

**Conclusion:**

This study presents a robust and interpretable ML-based approach for NPC risk prediction, integrating EBV serology and hematological markers. The model demonstrated high predictive accuracy and potential for population-based screening, providing an efficient tool for early NPC detection and intervention planning.

## 1 Introduction

Nasopharyngeal carcinoma (NPC) is a malignant tumor originating in the nasopharynx, an anatomical region located posterior to the nasal cavity and superior to the oropharynx (1). Although NPC is relatively rare in Western countries, it exhibits a markedly elevated incidence in certain parts of Asia, particularly Southeast Asia and Southern China (2), where it constitutes a significant public health concern (1). Specific geographic regions, such as Guangdong Province in southern China, demonstrate some of the highest incidence rates globally (3). Considerable variation in NPC risk has been observed among different ethnic subpopulations within Asia (4), including genetic susceptibility (1, 5–9), Epstein-Barr virus (EBV) infection (1, 5, 7, 10–12), environmental and lifestyle factors (1, 3, 7, 13), and socioeconomic and demographic factors (1, 13, 14), which appear to be the principal drivers of NPC’s high incidence of NPC in certain Asian populations. Therefore, understanding how genetic variability modulate EBV susceptibility and NPC development could pave the way for personalized risk assessment and preventive measures.

Many cases are diagnosed at advanced stages due to the asymptomatic nature of the condition in its early stages, which limits treatment options and leads to poor prognoses. The deep anatomical location of the nasopharynx, coupled with the nonspecific nature of early symptoms such as mild nasal congestion, headaches, or neck swelling, often delays diagnosis of NPC. Given these challenges, routine screening is crucial for improving early detection, facilitating timely intervention, and enhancing clinical outcomes (15).

Current screening approaches for NPC include serological biomarker detection, imaging techniques, and nasal endoscopy. Epstein-Barr virus (EBV) deoxyribonucleic acid (DNA) levels (8), along with virus capsid antigen-immunoglobulin A (VCA-IgA) and early antigen-immunoglobulin A (EA-IgA) antibodies, are widely recognized as key biomarkers for NPC risk assessment, offering a noninvasive approach to early detection (10, 16). However, alternative screening technologies, such as polarographic analysis, have been explored for their potential in detecting biochemical changes associated with NPC (17). Polarography, an electrochemical technique, has been employed to assess oxidative stress markers (18, 19), metabolic alterations (20, 21), and tumor-related enzymatic changes in biological fluids (22, 23), and may serve as an additional biomarker for NPC screening.

Studies have shown that the polarographic reduction of metal ions and nitro compounds can help identify redox activity changes in cancerous cells, potentially aiding in NPC detection. Ghorbian and Ghorbian (2023) reported that electrochemical signals in blood samples from patients with NPC exhibited distinct redox behaviors compared to those of healthy individuals, suggesting that polarographic methods could complement existing serological screening techniques (24). Furthermore, polarographic detection of EBV-associated metabolic changes may enhance the sensitivity and specificity of NPC risk assessment models, providing a novel avenue for noninvasive diagnostics (25).

Given the growing role of machine learning (ML) in NPC prediction, integrating polarographic biomarker analysis with traditional serological and epidemiological data could further refine risk stratification models (26). This study aimed to develop a comprehensive predictive model that incorporates EBV-DNA, IgA antibodies, smoking history, family history of NPC, and emerging electrochemical screening techniques to enhance early NPC detection and support clinical decision making (27).

A major challenge in NPC diagnosis is the reliance on nasal endoscopy and biopsy, which are invasive procedures typically performed only in symptomatic individuals. However, serological markers such as EBV-DNA and IgA antibodies (VCA-IgA and EA-IgA) have emerged as promising noninvasive biomarkers for early risk assessment (11, 28, 29). Studies have demonstrated that elevated EBV-DNA levels strongly correlate with NPC development; however, their clinical utility remains underutilized, particularly in primary healthcare settings. Additionally, lifestyle factors such as smoking, genetic predisposition, and environmental exposure influence NPC risk. However, their precise contributions require further quantification through predictive modeling (30).

Despite advancements in NPC screening, no widely adopted risk stratification model currently integrates serological, clinical, and epidemiological variables to guide early interventions. Existing screening strategies often lack sensitivity and specificity, resulting in missed cases or unnecessary invasive procedures (31). Developing an accurate and clinically interpretable NPC risk prediction model could significantly enhance screening protocols by identifying high-risk individuals who would benefit the most from further diagnostic testing, including endoscopy and imaging (30).

Accurate risk prediction is essential for the early detection of NPC, timely intervention, and improved patient outcomes. Prognosis for early stage NPC is generally favorable, with significantly higher survival rates compared to advanced-stage disease. Risk prediction models are deigned to identify individuals at elevated risk, thereby facilitating targeted screening and preventive counseling efforts. To address this gap, our study leveraged machine learning (ML) to develop a predictive model for NPC risk assessment. By utilizing least absolute shrinkage and selection operator (LASSO) regression for feature selection and logistic regression for modeling, we analyzed a comprehensive dataset of patients from diverse healthcare settings (32). This approach aims to identify key risk predictors and provide clinicians with a practical, data-driven screening tool to facilitate early NPC detection. Additionally, the integration of decision curve analysis (DCA) enhances the clinical relevance of the model by assessing its net benefit in guiding clinical decision-making. Through this research, we aimed to bridge the gap in NPC early screening, offering a robust framework for risk stratification, improved diagnostic precision, and timely intervention.

## 2 Materials and methods

### 2.1 Study design and eligibility criteria

The present retrospective study was conducted to develop and validate a ML-based predictive model for the early screening and risk assessment of NPC. A total of 1,000 individuals were initially enrolled for internal training and validation cohorts from The People’s Hospital of Guangxi Zhuang Autonomous Region, between January 2018 and June 2022. The study population included individuals undergoing routine health checkups, those presenting with NPC-related symptoms, and high-risk individuals, such as those with a family history of NPC or heavy smoking habits.

Participants were included if they met the following criteria: (1) aged ≥ 18 years, (2) provided informed consent for data collection and analysis, and (3) had complete clinical and serological records. The exclusion criteria were as follows: (1) a prior diagnosis of NPC, (2) the presence of severe comorbid conditions (e.g., advanced cancer, chronic liver disease, or autoimmune disorders), and (3) incomplete or missing key clinical data. After applying the inclusion and exclusion criteria, 431 participants were excluded, resulting in a final study population of 569 participants in the internal training and validation cohorts. The external validation cohort included 160 participants. Wuzhou Red Cross Hospital, the First People’s Hospital of Fangchenggang city, and the People’s Hospital of Yongning District, Nanning, between October 2022 to December 2024.

### 2.2 Data collection

Participant were categorized into two groups based on diagnostic findings: NPC (n = 234, 41.12%) and non-NPC (n = 335, 58.88%). Demographic data, including age and gender were recorded for all participants. Clinical assessments included EBV-related antibodies (nuclear antigen 1 immunoglobulin A (NTA1), Rta protein immunoglobulin A (Rta-IgA), viral capsid antigen immunoglobulin A (VCA-IgA), early antigen immunoglobulin A (EA-IgA), Zta protein immunoglobulin A (Zta-IgA)), liver function tests (alanine aminotransferase (ALT), aspartate aminotransferase (AST), total protein (TP), albumin (ALB), total bilirubin (TB), direct bilirubin (DB), cholinesterase (CHE), adenosine deaminase (ADA), alkaline phosphatase (ALP), total bile acid (TBA), gamma-glutamyl transferase (GGT), prealbumin (PA)), renal function markers (urea, creatinine (CREA), uric acid (UA)), glucose and lipid metabolism (glucose (GLU), triglycerides (TG), high density lipoprotein (HDL), low density lipoprotein (LDL), apolipoprotein A-I (APOPA1), apolipoprotein B (APOB), lipoprotein A (LPA)), and hematology (white blood cell (WBC), red blood cell (RBC), hemoglobin (HB), hematocrit (HCT), mean corpuscular volume (MCV), mean corpuscular hemoglobin (MCH), platelet count (PLT), red cell distribution width (RDW), platelet distribution width (PDW), and lymphocyte (LM)). Feature selection was performed using least absolute shrinkage and selection operator (LASSO) regression, recursive feature elimination cross-validation (REFCV), and support vector machine recursive feature elimination cross-validation (SVMREFCV).

### 2.3 Machine learning

To identify significant predictors, least absolute shrinkage and selection operator (LASSO) regression was applied using the ‘glmnet’ package in R. This method filtered out irrelevant or redundant features, retaining only the most informative variables for NPC risk prediction. Following feature selection, predictive models were developed using both logistic regression and multiple machine learning (ML) algorithms. The dataset was randomly split into a training set (70%) and a validation set (30%) using RStudio (version 2025.05.0 + 496) with the ‘ggplot2’ package. Although the overall class distribution between the NPC and non-NPC participants was moderately imbalanced (41.12% vs 58.88%), model robustness was maintained by employing class-weight adjustments during training to ensure balanced learning across classes. Sensitivity and specificity were jointly evaluated to assess the balanced performance. In future studies, advanced resampling methods, such as the Synthetic Minority Oversampling Technique (SMOTE) and stratified cross-validation, will be explored to further mitigate potential class imbalance effects. The following ML models were evaluated: logistic regression (for interpretability and clinical application), eXtreme Gradient Boosting (XGBoost), light gradient boosting machine (LightGBM), random forest (RF), decision tree (DT), AdaBoost, multilayer perceptron (MLP), Gaussian Naïve Bayes (GNB), and gradient boosting decision tree (GBDT). Model calibration was conducted, and predictive performance was evaluated using area under the curve (AUC), accuracy (ACC), sensitivity (SE), and specificity (SP) in both training and validation cohorts. All Statistical analyses were performed using R version 4.2.3 and python version 3.11.4.

### 2.4 Sample size and power analysis

Our primary performance metric was the area under the ROC curve (AUC). A *post hoc* power analysis (two-sided α = 0.05) using R indicated that the internal cohort (n = 569; NPC cases = 234, non-NPC = 335) provides >99% power to detect an AUC ≥ 0.80 versus the null hypothesis AUC = 0.50. The independent external cohort (n = 160; NPC = 67, non-NPC = 93) provides ~95% power to detect an AUC ≥ 0.80 versus AUC = 0.50. In addition, model complexity was constrained relative to events: with five final predictors, the events-per-variable (EPV) was ~47 (234/5), exceeding conventional EPV recommendations (≥10–20) for logistic/ML models and reducing the risk of overfitting. For reproducibility, we fixed the random seed (set.seed(1234)) and reported the package versions and key hyperparameters in Section 2.3. In future prospective studies, we plan to determine the sample size *a priori* based on the anticipated AUC and desired confidence interval width to pre-specify precision.

### 2.5 Statistical analysis

Statistical analyses and ML model training were performed using RStudio (version 2025.05.0 + 496) with the ggplot2 package. Descriptive statistics were reported as means ± standard deviations (SD) for continuous variables and proportions for categorical variables. Between-group comparisons were conducted using intendent t-tests for continuous variables and chi-square tests for categorical variables. Statistical significance was set at p < 0.05.

## 3 Results

### 3.1 The baseline characteristics of enrolled participants

A total of 569 participants were included in the internal training and validation cohort, comprising 234 (41.12%) individuals in the NPC-positive group and 335 (58.88%) in the non-NPC group. All five EBV-related antibodies (NTA1, Rta-IgA, VCA-IgA, EA-IgA, and Zta-IgA) showed statistically significant differences between the two groups (p < 0.001). Similarly, the levels of total protein (TP), albumin (ALB), prealbumin (PA), and uric acid (UA) differed significantly (p < 0.001). Additional biomarkers, including HDL, APOA1, APOB, RBC, HB, HCT, PLT, RDW, and LM, also demonstrated significant differences between the two groups (p < 0.001) (**Table 1**).

**Table 1:** The baseline clinical characteristics of included patients.

Characteristics	Non-NPC	NPC	P-value
Participants (n, %)	335	234	
Gender (n, %)			< 0.001
Male	191	90	
Female	144	143	
Age (mean ± SD)	47.81 ± 14.88	48.37 ± 11.52	0.631
EBV-related antibodies (mean ± SD)			
Nuclear antigen 1 immunoglobulin A (NTA1)	0.14 ± 0.48	6.41 ± 4.66	< 0.001
Rta protein immunoglobulin A (Rta-IgA)	0.40 ± 0.27	3.64 ± 3.81	< 0.001
Viral capsid antigen immunoglobulin A (VCA-IgA)	0.26 ± 0.40	4.75 ± 2.72	< 0.001
Early antigen immunoglobulin A (EA-IgA)	0.20 ± 0.55	2.02 ± 2.17	< 0.001
Zta protein immunoglobulin A (Zta-IgA)	0.38 ± 0.55	1.75 ± 1.38	< 0.001
Liver function tests (mean ± SD)			
Alanine aminotransferase (ALT)	22.68 ± 17.64	27.01 ± 34.62	0.052
Aspartate aminotransferase (AST)	25.04 ± 8.87	26.59 ± 25.24	0.302
Total protein (TP)	73.70 ± 5.24	69.45 ± 5.33	< 0.001
Albumin (ALB)	43.17 ± 4.60	39.67 ± 3.33	< 0.001
Total bilirubin (TB)	12.50 ± 5.11	12.75 ± 6.43	0.602
Direct bilirubin (DB)	2.21 ± 0.88	2.28 ± 1.14	0.425
Cholinesterase (CHE)	8772.98 ± 1736.48	8162.96 ± 5151.10	0.046
Adenosine deaminase (ADA)	10.99 ± 6.30	9.51 ± 4.42	0.002
Alkaline phosphatase (ALP)	72.82 ± 26.57	82.18 ± 71.45	0.029
Total bile acid (TBA)	4.83 ± 6.70	6.19 ± 8.29	0.033
Gamma-Glutamyl Transferase (GGT)	34.52 ± 28.41	33.37 ± 32.28	0.654
Prealbumin (PA)	279.04 ± 55.92	240.76 ± 64.79	< 0.001
Renal function tests (mean ± SD)			
UERA	5.33 ± 2.66	6.41 ± 24.35	0.423
Creatinine (CREA)	78.37 ± 50.98	74.93 ± 19.31	0.343
Uric acid (UA)	394.44 ± 101.51	346.29 ± 93.73	< 0.001
Glucose and lipid metabolism (mean ± SD)			
Glucose (GLU)	4.76 ± 1.54	5.06 ± 1.93	0.044
Triglycerides (TG)	3.10 ± 23.91	1.38 ± 0.69	0.383
High density lipoprotein (HDL)	1.30 ± 0.33	1.09 ± 0.30	< 0.001
Low density lipoprotein (LDL)	3.20 ± 0.77	3.08 ± 0.81	0.111
Apolipoprotein A-I (APOA1)	1.48 ± 0.29	1.08 ± 0.31	< 0.001
Apolipoprotein B (APOB)	1.03 ± 0.29	0.92 ± 0.29	< 0.001
Lipoprotein A (LPA)	207.65 ± 273.94	198.09 ± 212.91	0.712
Hematology (mean ± SD)			
White blood cell (WBC) count	8.75 ± 40.60	7.59 ± 2.95	0.703
Red blood cell (RBC) count	4.96 ± 0.68	4.70 ± 0.75	< 0.001
Hemoglobin (HB)	140.93 ± 15.71	130.36 ± 21.07	< 0.001
Hematocrit (HCT)	42.96 ± 4.16	39.94 ± 5.86	< 0.001
Mean corpuscular volume (MCV)	87.36 ± 8.40	85.59 ± 9.64	0.031
Mean corpuscular hemoglobin (MCH)	28.69 ± 3.37	28.13 ± 4.44	0.111
Platelet count (PLT)	251.14 ± 58.68	287.48 ± 74.32	< 0.001
Red cell distribution width (RDW)	35.38 ± 83.69	40.93 ± 4.70	< 0.001
Platelet distribution width (PDW)	12.41 ± 10.01	10.28 ± 1.52	0.005
Lymphocyte (LM)	5.12 ± 1.75	3.21 ± 2.74	< 0.001

### 3.2 Feature selection correlated with NPC

**Figure 1:**
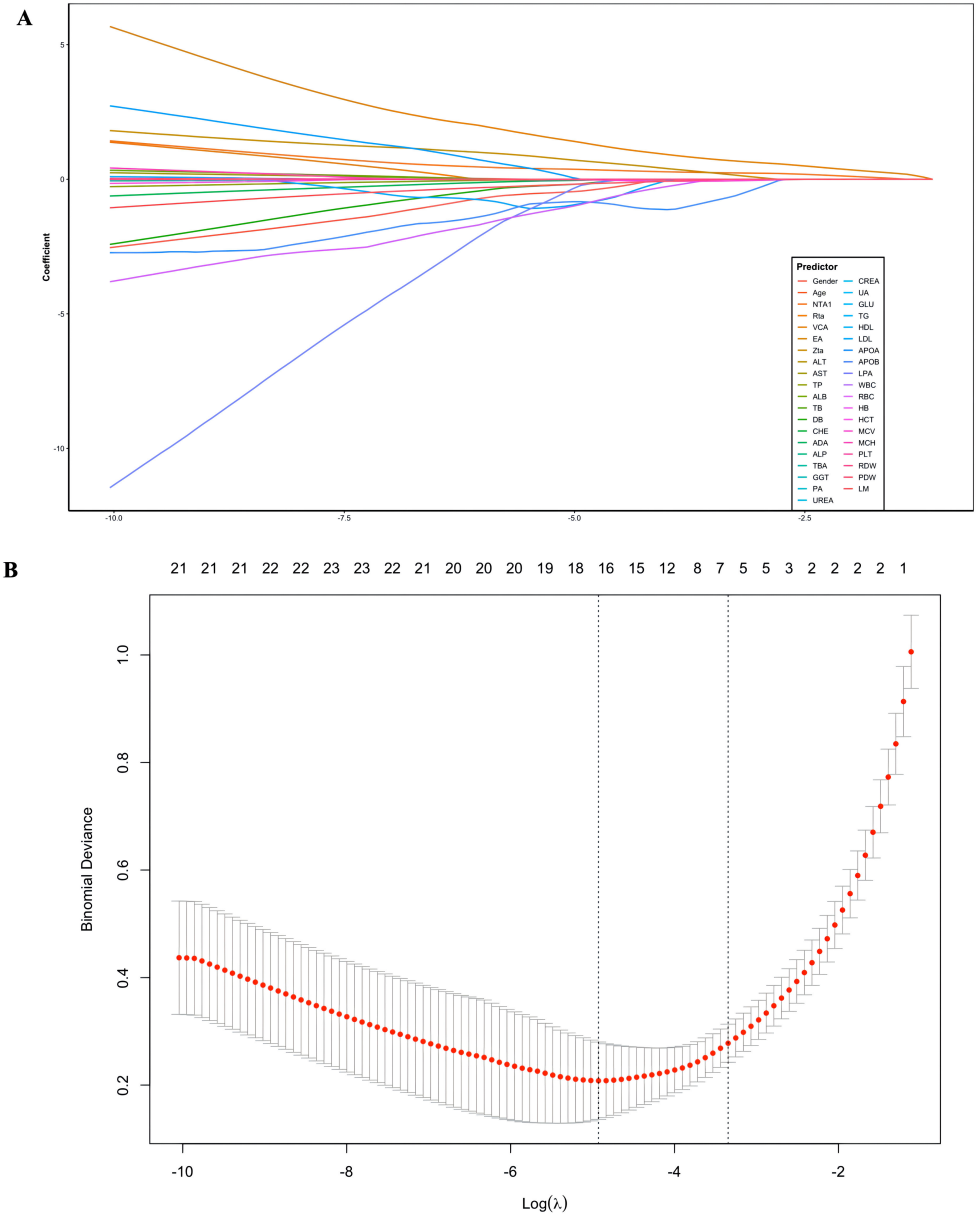
Least absolute shrinkage and selection operator (LASSO) regression analysis and 10-fold cross-validation for selecting factors associated with NPC. **(A)** LASSO coefficient path; **(B)** LASSO cross-validation curve.

**Figure 2:**
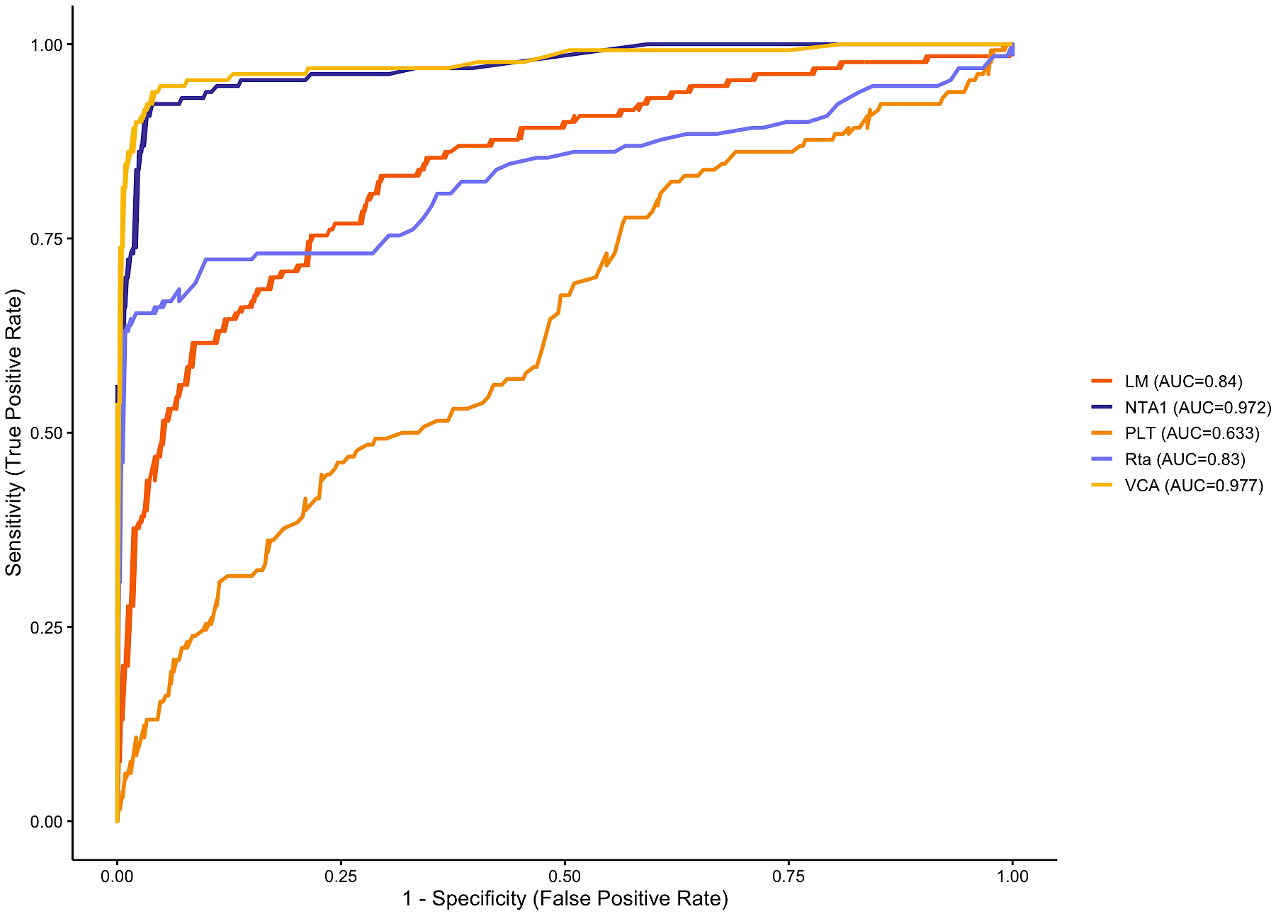
ROC curves for the 5 selected predictors: NTA1, Rta, VCA, PLT, and LM.

Key predictor associated with NPC risk were identified using LASSO regression combined with 10-fold cross-validation (**Figure 1**). The most relevant predictors included NTA1, VCA, Rta, PLT, and LM. ROC analysis demonstrated that VCA had the highest AUC value (0.977), followed by NTA1 (AUC = 0.972). PLT had the lowest AUC value (0.633). While Rta (AUC = 0.83) and LM (AUC = 0.84) had moderate AUC values (**Figure 2**).

### 3.3 Features identified using ML algorithms

**Figure 3:**
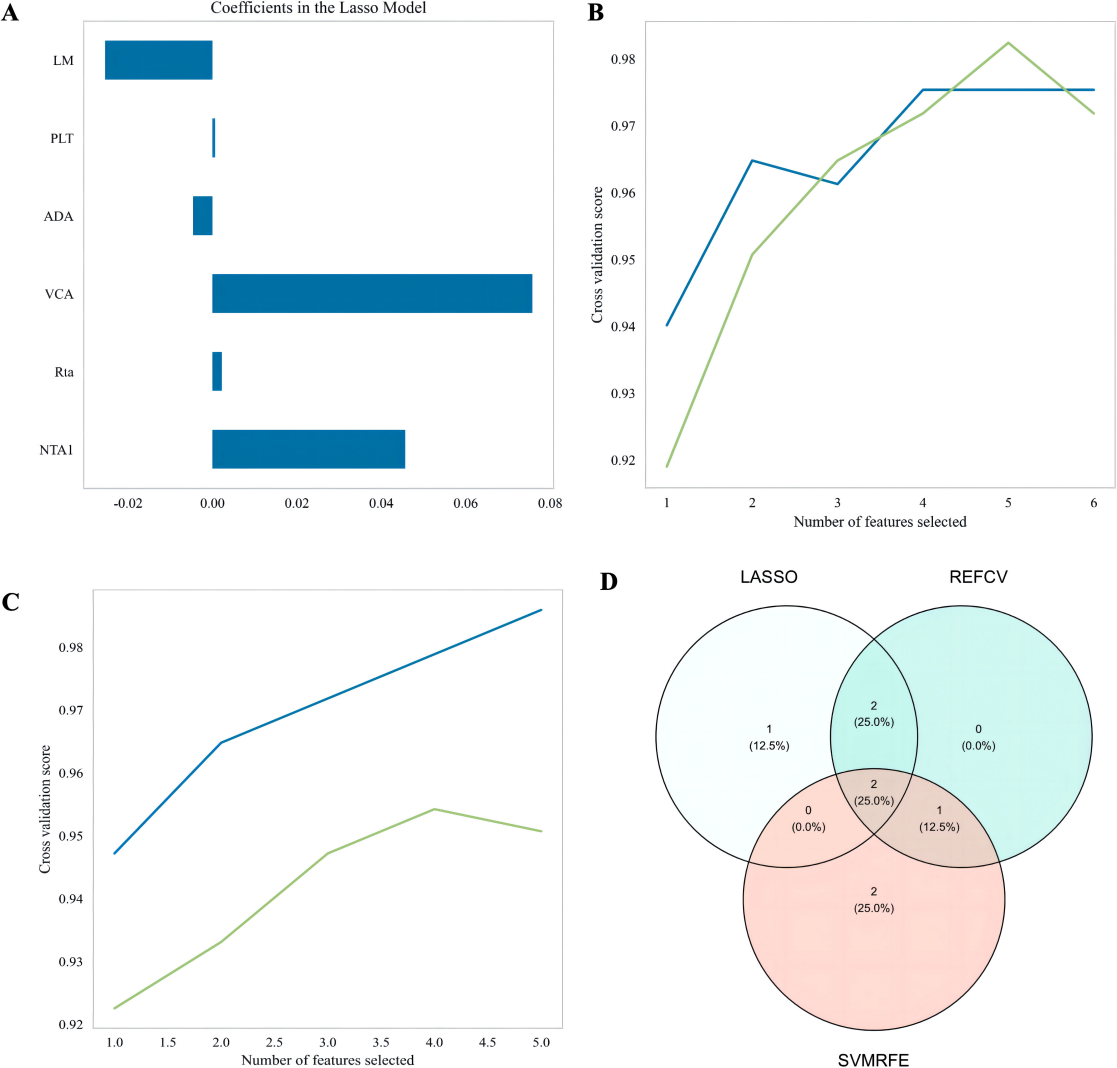
Identification of characteristic markers. **(A)** Six markers were identified using the LASSOCV algorithm; **(B)** five markers were identified using the repeated elastic net feature Cross-validation (REFCV) algorithm; **(C)** five markers were identified using the support vector machine recursive feature elimination with cross-validation (SVMREFCV) algorithm; **(D)** Venn plot of markers for three machine-learning algorithms.

Three ML algorithms - LASSOCV, REFCV, and SVMREFCV - were used to identify biomarkers associated with NPC (**Figures 3A–C**, respectively). A Veen diagram was generated using RStudio to illustrate the overlapping features (**Figure 3D**). The five features (NTA1, VCA, Rta, PLT, and LM) were consistently selected across methods, and the obtained results showed that they were the most associated factors with NPC risk.

### 3.4 Model evaluations

**Figure 4:**
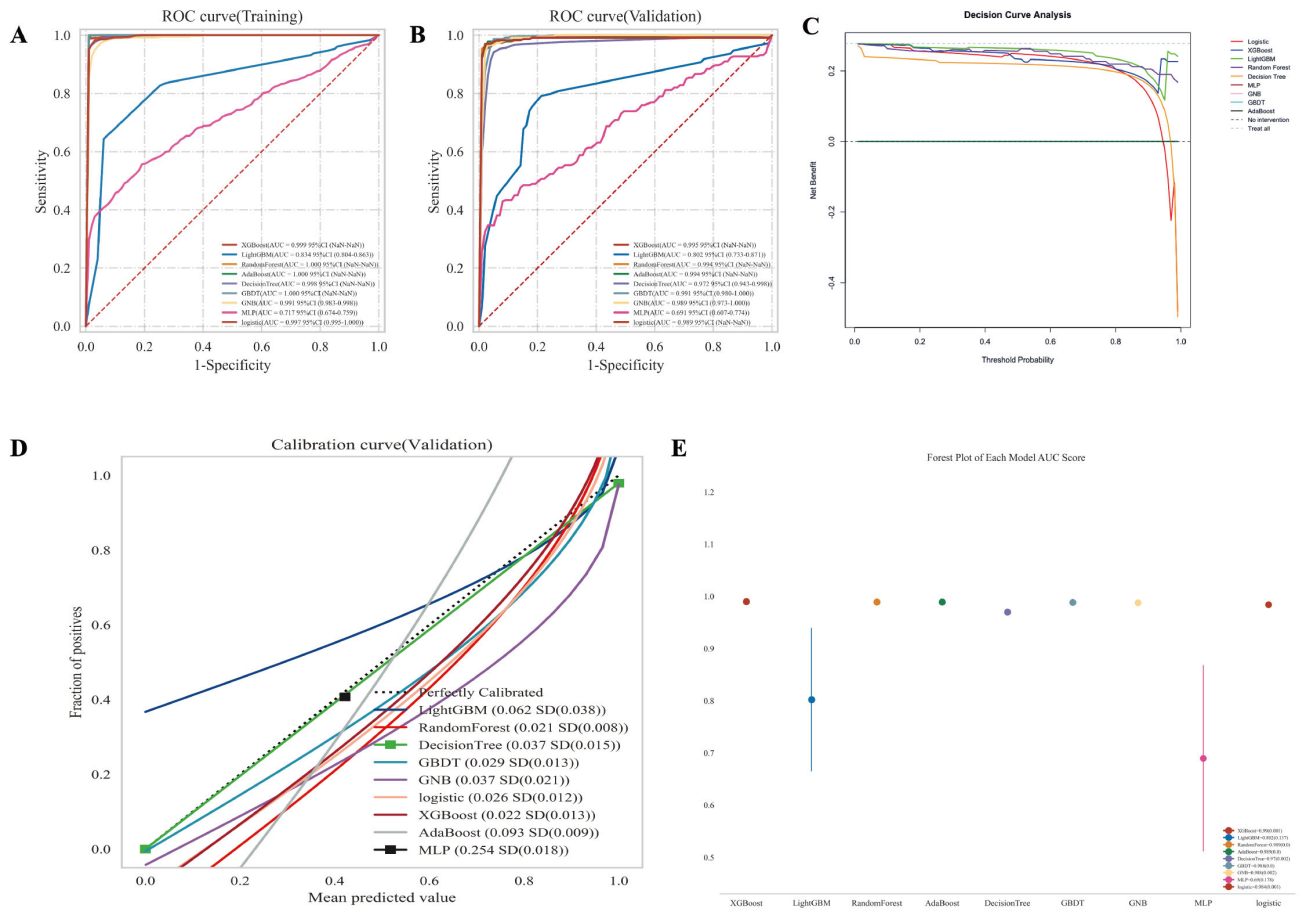
Performance comparison between multiple models. **(A)** Receiver operating characteristic (ROC) curve of the training cohort; **(B)** ROC curve of the validation cohort; **(C)** decision curve of multiple machine-learning (ML) models; **(D)** calibration curve of different ML models; **(E)** forest plot of each area under the curve (AUC) score.

**Figure 5:**
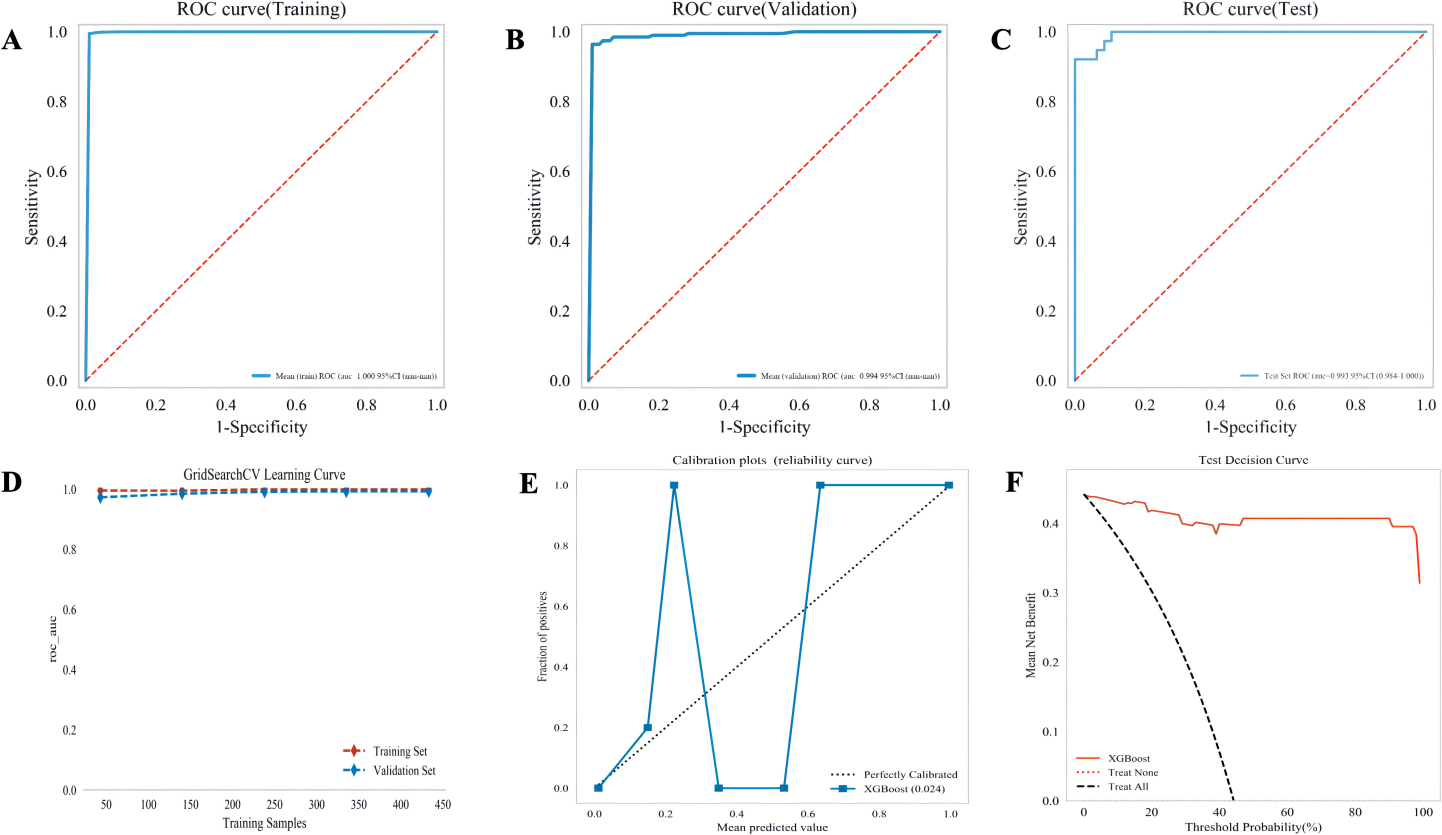
Performance of the XGBoost prediction model across cohorts**. (A–C)** Receiver operating characteristic (ROC) curves for the training, validation, and testing cohorts, respectively, showing sensitivity vs. 1 specificity. **(D)** GridSearchCV learning curve illustrating AUC convergence for training and validation sets across sample sizes. **(E)** Calibration (reliability) curve comparing predicted probabilities with observed outcomes; dashed line indicates perfect calibration. **(F)** Decision curve analysis (DCA) demonstrating the net clinical benefit across threshold probabilities. All axes are consistently labeled; units represent proportions or probabilities (0–1).

The predictive performance of nine ML models was assessed in both training and validation cohorts (**Table 2**; **Figure 4**). The XGBoost model demonstrated superior performance, achieving an AUC of 0.999, sensitivity of 0.985, and specificity of 0.999 in the training cohorts. In the validation cohort, the XGBoost model maintained the highest AUC (0.995), ACC (0.959), SE (0.94), and SP (0.973). Calibration plots and decision curve analysis further supported the outstanding performance and clinical utility of the XGBoost model. Further analysis demonstrated the robust generalization of the XGBoost model. As shown in **Figures 5A–D**, the AUC values for the test cohort (AUC = 0.993) and validation cohort (AUC = 0.994) were slightly lower than the training cohort (AUC = 1.000), suggesting high model fidelity. Although these near-perfect AUC values indicate excellent discriminative ability, they may also reflect potential overfitting in the model. To minimize this risk, we applied rigorous feature selection (LASSO, REFCV, and SVMREFCV), used an independent external validation cohort, and conducted calibration and decision curve analyses to evaluate model reliability. Calibration plot and decision curve analysis of the XGBoost model are shown in **Figures 5E, F**, further confirmed the strong fitting ability and high clinical utility of the XGBoost model.

**Table 2:** Diagnostic efficacy of nine classifiers in the internal training and validation cohorts.

Models	Cohorts	AUC	Cutoff	Accuracy	Sensitivity	Specificity	Positive predictive value	Negative predictive value	F1
XGBoost|	Training	0.999	0.43	0.993	0.985	0.999	0.998	0.99	0.991
	Validation	0.995	0.43	0.959	0.94	0.973	0.961	0.959	0.95
LightGBM	Training	0.834	0.77	0.863	0.783	0.919	0.902	0.894	0.771
	Validation	0.802	0.77	0.838	0.735	0.91	0.888	0.864	0.735
Random Forest	Training	1.000	0.6	1.000	1.000	1.000	1.000	1.000	1.000
	Validation	0.994	0.6	0.974	0.944	0.994	0.991	0.963	0.967
AdaBoost	Training	1.000	0.493	0.997	0.999	0.996	0.995	0.999	0.997
	Validation	0.994	0.493	0.981	0.966	0.991	0.987	0.977	0.976
Decision tree	Training	0.998	0.827	0.981	0.973	0.987	0.981	0.982	0.977
	Validation	0.972	0.827	0.961	0.953	0.967	0.955	0.967	0.953
GBDT	Training	1.000	0.998	1.000	1.000	1.000	1.000	1.000	1.000
	Validation	0.991	0.998	0.963	0.931	0.985	0.979	0.955	0.954
GNB	Training	0.991	0.032	0.964	0.974	0.957	0.941	0.982	0.957
	Validation	0.989	0.032	0.96	0.966	0.955	0.939	0.976	0.952
MLP	Training	0.717	0.452	0.726	0.606	0.810	0.686	0.759	0.618
	Validation	0.691	0.452	0.694	0.589	0.767	0.645	0.74	0.587
Logistic regression	Training	0.997	0.346	0.98	0.977	0.981	0.974	0.984	0.975
	Validation	0.989	0.346	0.963	0.953	0.97	0.959	0.969	0.955

AUC, Area under the curve; XGBoost, Extreme gradient boosting; LightGBM, Light gradient boosting machine; AdaBoost, Adaptive boosting; GBDT, Gradient boosted decision tree; GNB, Gaussian naïve bayes; MLP, Multilayer perceptron.

### 3.5 External validation of the XGBoost model

**Figure 6:**
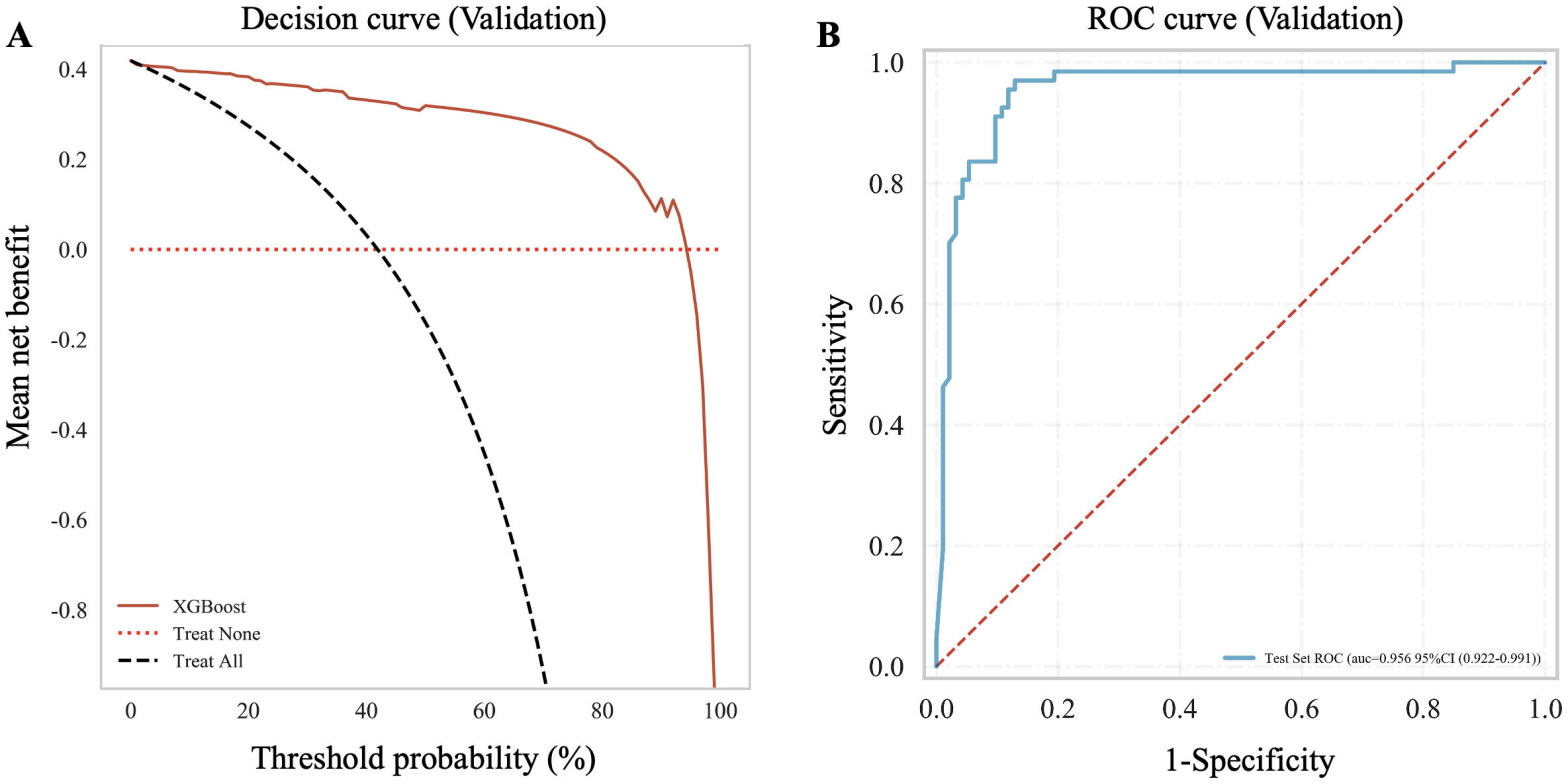
Performance of the XGBoost model in the external validation cohort. **(A)** Decision curve analysis (DCA) illustrating the net clinical benefit of the XGBoost model compared with “Treat None” and “Treat All” strategies across threshold probabilities (%). **(B)** Receiver operating characteristic (ROC) curve showing model discrimination with an AUC of 0.956 (95% CI 0.922–0.991); axes display sensitivity vs. 1 – specificity.

A total of 160 participants were included in the independent external validation cohort from three other centers. It compromised of 93 (58.13%) individuals in non-NPC group and 67 (41.88%) in NPC group (**Table 3**). The XGBoost model maintained the highest predictive performance with an AUC of 0.956 (**Table 4**; **Figure 6**), and the decision curve indicated a strong clinical benefit (**Figure 6**).

**Table 3:** Characteristics of external validation cohort.

Biomarkers	Non-NPC	NPC	P-value
NTA1	0.25 ± 0.79	4.55 ± 3.88	0.000
Rta-IgA	0.64 ± 0.76	1.81 ± 1.33	0.000
VCA-IgA	0.58 ± 0.60	1.92 ± 1.14	0.000
PLT	271.63 ± 56.95	243.76 ± 76.19	0.009
LM	5.56 ± 1.91	2.56 ± 1.31	0.000

NTA1, Nuclear antigen 1 immunoglobulin A; Rta-IgA, Rta protein immunoglobulin A; VCA, Viral capsid antigen immunoglobulin A; PLT, Platelet count; LM, Lymphocyte.

**Table 4:** Diagnostic efficacy of nine classifiers in the external validation cohorts.

Model	Cohorts	AUC	Cutoff	Accuracy	Sensitivity	Specificity	Positive predictive value	Negative predictive value	F1
XGBoost	Validation	0.956	0.381	0.894	0.881	0.903	0.868	0.913	0.874

XGBoost, Extreme gradient boosting.

### 3.6 Model interpretation with SHAP

**Figure 7:**
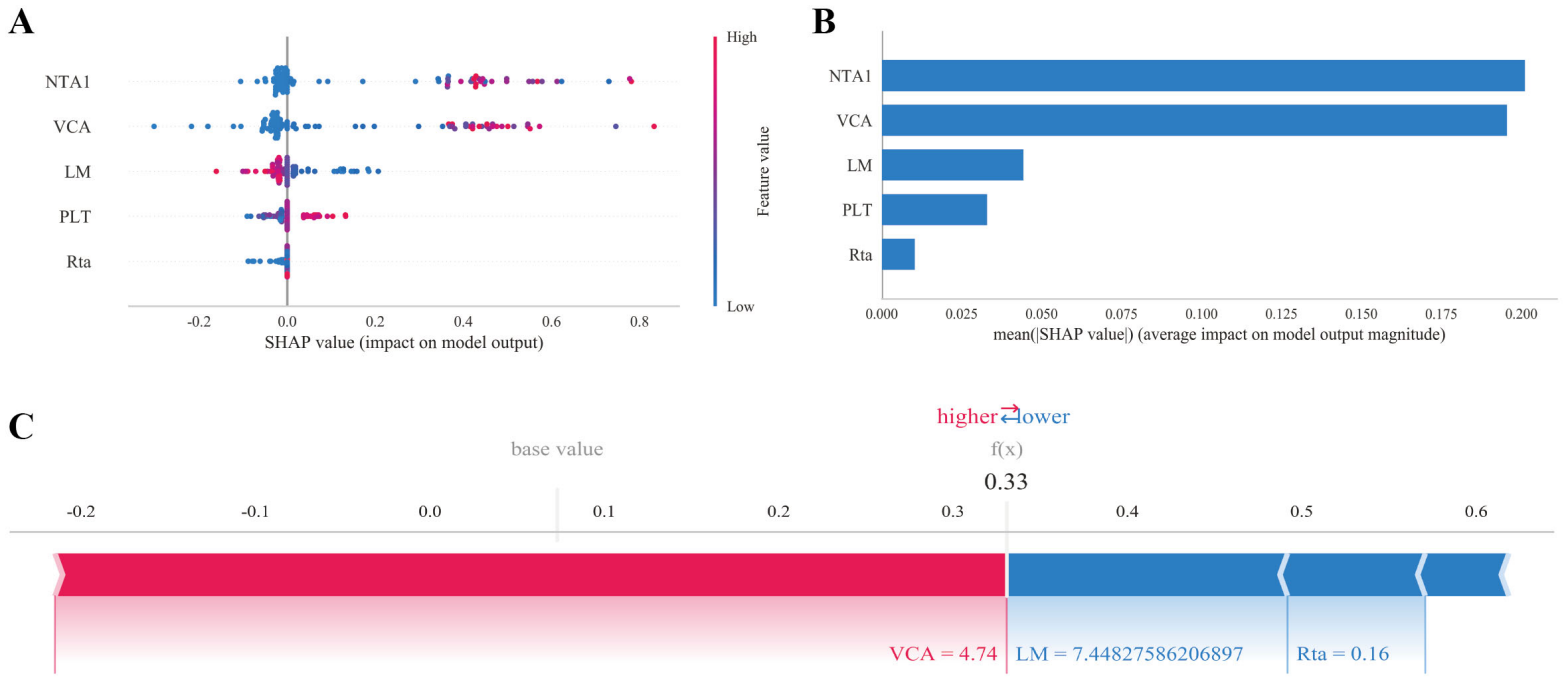
SHAP-based interpretability of the XGBoost model. **(A)** Global Mean Absolute SHAP Values Plot. **(B)** Feature Importance Bar Chart. **(C)** Dependence Plot for an Exemplary High-Risk Participant.

To quantify the contribution of each biomarker to the XGBoost model, SHAP (SHapley Additive exPlanations) values were computed for all participants in the validation cohort. The global mean SHAP plot (**Figures 7A, B**) ranked VCA-IgA and NTA1-IgA as the two most influential features, followed by lymphocyte counts, platelet and Rta-IgA. Dependence plots revealed clear dose–response relationships: higher VCA-IgA titers systematically increased the predicted probability, whereas elevated lymphocyte counts and Rta-IgA exerted a protective effect. A dependence plot for an exemplar high-risk participant (**Figure 7C**) showed that the VCA-IgA antibodies alone contributed +4.74 log-odds, respectively, accounting for ~75% of the final risk score. These analyses confirm that the model relies on biologically plausible drivers and provide clinician-readable explanations for each prediction.

### 3.7 Online prediction tool

**Figure 8:**
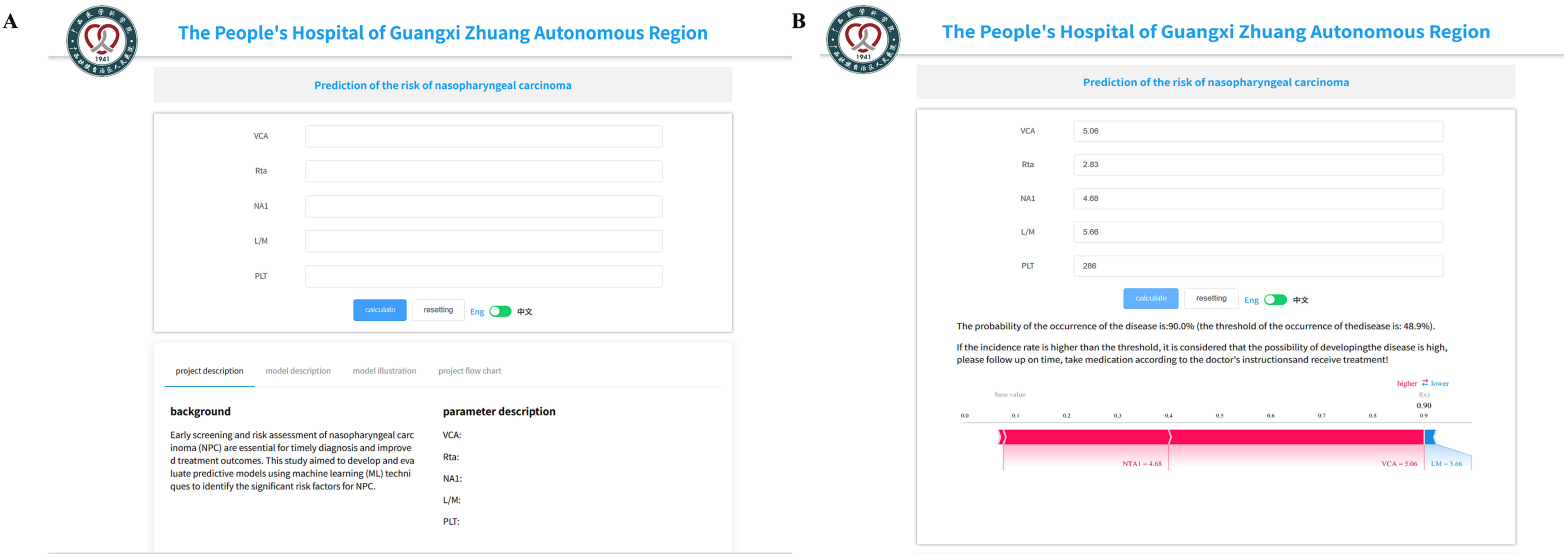
An online prediction tool to predict the risk of mortality. **(A)** An online page based on the XGBoost algorithm. **(B)** An online page to predict the risk of NPC based on five predictors.

An online prediction tool was also developed to facilitate clinician-friendly interpretation of NPC risk (*https://www.xsmartanalysis.com/model/list/predict/model/html?mid=25653&symbol=51PY7477364ykEV6Jk11*). The five selected predictors (NTA1, VCA, Rta, PLT, and LM) were used as input variables. Model explainability was enhanced using SHAP values to quantify the individual contribution of each predictor to the overall model performance. VCA (SHAP = 5.06) and NTA1 (SHAP = 4.68) exerted the strongest positive influence on NPC risk, reflecting their critical diagnostic relevance, whereas LM and PLT contributed modestly, suggesting an auxiliary role in the systemic immune response. The integration of SHAP visualization within the online interface allows clinicians to intuitively interpret how each biomarker drives individual risk predictions (**Figures 8A, B**).

## 4 Discussion

The present study leveraged a large-scale cohort of 569 participants and integrated serological and hematological markers with advanced ML techniques to develop a high-performing model for NPC risk prediction. The model development process involved multiple stages, including LASSO analysis, feature selection, ROC analysis, model evaluation, calibration plots and decision curve analysis, to demonstrate the efficacy of the nine ML models (logistic regression, XGBoost, LightGBM, RF, DT, AdaBoost, MLP, GNB, and GBDT) in identifying key risk factors for NPC and in providing a reliable, clinically applicable tool for early detection and decision-making across varying healthcare settings.

Through LASSO, REFCV, and SVMREFCV analysis, we selected five predictors: three EBV-related antibodies (NTA1, VCA, and Rta), which reinforce the central role of EBV serology in NPC pathogenesis, and two hematological indicators (PLT and LM), reflecting host immune and inflammatory dynamics (**Figures 1**, **3**). To enhance interpretability and clinical usability, we further employed SHAP (SHapley Additive exPlanations) analysis to quantify each feature’s contribution to model output. The results (**Figure 7C**) indicated that VCA and NTA1 exhibited the strongest positive influence on NPC risk prediction, whereas LM,PLT and Rta contributed modestly but consistently to the overall model discrimination. These insights help bridge the gap between algorithmic prediction and clinical reasoning, enabling end-users to understand how each variable drives individual risk estimates. More importantly, we found that VCA and NTA1 had the highest AUC values in the ROC analysis, indicating that VCA and NTA1 are the most relevant predictors of NPC risk. These results are consistent with previous research highlighting the role of EBV-related antibodies, especially VCA-IgA and NTA1, in the pathogenesis and early detection of NPC (25, 33, 34). Rta-IgA also further corroborates the role of EBV reactivation in NPC development, as Rta is a lytic protein expressed during EBV replication (10, 35). In addition to serological markers, although PLT had the lowest AUC values, we observed that hematological parameters such as PLT and LM were still significantly associated with NPC risk. These findings are consistent with Wu et al. (36), who first constructed a predictive model using baseline LM subpopulations to estimate immunotherapy responses in NPC patients (36), our findings further support the critical role of systemic inflammation and immune dynamics in NPC progression and prognosis. Moreover, these findings are in line with prior work by Long et al. (37), who incorporated blood-based markers into ML development and demonstrated that including the platelet-lymphocyte ratio (PLR), particularly within a logistic regression framework, significantly enhanced predictive performance and clinical utility (37). These insights are crucial as they guide clinicians in identifying high-risk individuals, particularly those who may benefit from more intensive screening, and emphasized the importance of ML models in clinical practice (38).Thus, SHAP transforms the ensemble model into a transparent, instance-level decision aid that can be displayed alongside the risk score, increasing clinician trust and facilitating patient counseling.

Moreover, among the nine ML models evaluated, the XGBoost model consistently outperformed the others across all metrics, achieving an AUC of 1.000 in the training cohort and 0.995 in the internal validation cohort (**Table 2**), as well as 0.956 in the independent external validation cohort (**Table 4**). While these near-perfect AUC values highlight the strong discriminative ability of the model, they may also raise concerns regarding potential overfitting. To mitigate this risk and ensure generalizability, we adopted several strategies, including independent external validation, careful feature selection using three algorithms (LASSO, REFCV, and SVMREFCV) and model calibration assessment. In future studies, nested cross-validation, enhanced regularization tuning, and complexity reduction will be considered to further confirm the robustness of the XGBoost model. Decision curve analysis for both internal and external validation cohort also confirmed its clinical applicability, suggesting that the XGBoost-based model could be a valuable tool for early NPC screening and risk stratification in large populations. Our study highlights the utility of incorporating EBV-related serological markers, such as NTA1, Rta, and VCA, alongside hematological parameters (e.g., PLT and LM) for risk prediction in NPC. In contrast, Chen et al. (2024) developed a novel XGBoost model based on hospital electronic medical records (EMR) and the patient graph connection delta ratio (CDR), deliberately excluding EBV-related antibodies from their model construction process (39). Despite this exclusion, their model achieved strong predictive performance (AUC = 0.87) (39), suggesting they non-serological features derived from EMR and patient network structures can also effectively capture NPC risk. While another study conducted by Chen et al. (2024). However, our findings suggest that integrating virological biomarkers may provide added biological relevance and potentially improve early detection, especially in high-risk populations. The complementary nature of these approaches underscores the need for multi-modal data integration in NPC risk modeling. Future studies should explore hybrid models that combine clinical, serological, and network-based features to enhance predictive accuracy and clinical applicability across diverse healthcare settings. Furthermore, the implementation of an online prediction tool based on the five selected features (NTA1, Rta, VCA, PLT, and LM) offers a practical approach for clinicians to assess NPC risk in real time. To enhance interpretability and facilitate clinical adoption, SHAP-based model explainability was applied, enabling the visualization of each biomarker’s individual and combined influence on NPC risk. This approach bridges the gap between model accuracy and clinical transparency, allowing practitioners to better understand the biological rationale behind the predictions and make informed decisions. This tool could facilitate personalized screening strategies, especially in high-incidence regions, thereby improving early diagnosis and outcomes.

The model developed in this study holds significant promise for improving NPC risk prediction across a range of healthcare settings. By integrating clinical data and laboratory biomarkers, the model is adaptable to varying resource settings, from township hospitals to tertiary medical centers. This flexibility is essential, as it allows the model to be implemented in regions with differing levels of diagnostic infrastructure. Clinicians can use this tool to identify individuals at high risk of NPC, potentially reducing the need for invasive procedures such as Naso endoscopy in low-risk individuals. While the results are promising, there are several areas for future work. Further validation of the model in independent and multi-center cohorts is necessary to assess its generalizability across diverse populations. Although the present study focused primarily on serological and hematological predictors due to their accessibility and non-invasive nature, the absence of radiological data (e.g., CT, MRI, or PET imaging) represents a limitation. Integrating radiological or radiomic features in future hybrid models could significantly enhance diagnostic comprehensiveness by capturing both anatomical and molecular information. Such multimodal frameworks combining imaging, serological, and genomic data may improve predictive accuracy, interpretability, and clinical applicability. In addition, the inclusion of other potentially relevant biomarkers or clinical variables, especially in high-burden settings should be explored. The AdaBoost model was excluded from calibration analysis due to computational instability during curve generation; future studies should revisit its integration. Finally, embedding the XGBoost model within clinical decision support systems, along with real-time model updates, could further strengthen its role in NPC early detection and management. However, class imbalance in the dataset was moderate, class-weight adjustments were applied to counter the potential bias toward the majority group. Future work will systematically evaluate the impact of data balancing strategies such as SMOTE and stratified sampling to ensure equitable performance across classes.

## 5 Conclusion

In this large-scale study, we identified a sensitive panel of serological and hematological biomarkers – NTA1, VCA, Rta, PLT, and LM – associated with NPC risk. By integrating these predicators into the ML framework, we developed and validated an XGBoost-based predicted model that achieved near-perfect performance across internal training and validation cohorts, and external validation cohort. The XGBoost model’s accuracy, coupled with its clinical interpretability and integration into an online tool, offers a scalable solution for early NPC risk stratification. These findings underscore the potential of combining population-based serological profiling with ML to enhance NPC screening strategies. Prospective, multi-center validation is warranted to support clinical implementation.

## Data Availability

The original contributions presented in the study are included in the article/supplementary material. Further inquiries can be directed to the corresponding author.
